# Bereaved family members’ perspectives on quality of death in deceased acute cardiovascular disease patients compared with cancer patients – a comparison of the J-HOPE3 study and the quality of palliative care in heart disease (Q-PACH) study

**DOI:** 10.1186/s12904-024-01521-4

**Published:** 2024-07-26

**Authors:** Takahiro Suzuki, Mitsunori Miyashita, Takashi Kohno, Jeffrey Rewley, Naoko Igarashi, Maho Aoyama, Michiaki Higashitani, Naoto Kawamatsu, Takeshi Kitai, Tatsuhiro Shibata, Makoto Takei, Kotaro Nochioka, Gaku Nakazawa, Hiroki Shiomi, Shigeru Tateno, Toshihisa Anzai, Atsushi Mizuno

**Affiliations:** 1 Department of Cardiovascular Medicine, St. Luke’s International Hospital, Tokyo, Japan; 2 Division of Cardiology, Department of Medicine, Keio University School of Medicine, Tokyo, Japan; 3 Leonard Davis Institute of Health Economics, University of Pennsylvania, Philadelphia, PA USA; 4 Department of Palliative Nursing, Health Sciences, Tohoku University Graduate School of Medicine, Sendai, Miyagi Japan; 5 Department of Cardiology, Tokyo Medical University Ibaraki Medical Center, Ibaraki, Japan; 6 Department of Cardiology, Mito Saiseikai General Hospital, Mito, Japan; 7 Departments of Cardiovascular Medicine, Kobe City Medical Center General Hospital, Kobe, Japan; 8 Departments of Clinical Research Support, Kobe City Medical Center General Hospital, Kobe, Japan; 9 Division of Cardiovascular Medicine, Department of Internal Medicine, Kurume University School of Medicine, Kurume, Japan; 10 Department of Cardiology, Tokyo Saiseikai Central Hospital, Tokyo, Japan; 11 Department of Cardiovascular Medicine, Tohoku University Graduate School of Medicine, Sendai, Japan; 12 Department of Cardiology, Tokai University School of Medicine, Tokyo, Japan; 13 Department of Cardiovascular Medicine, Kyoto University Graduate School of Medicine, Kyoto, Japan; 14 Department of Pediatrics, Chiba Cerebral and Cardiovascular Center, Ichihara, Japan; 15 Department of Cardiovascular Medicine, Hokkaido University Graduate School of Medicine, Sapporo, Japan; 16 The MITRE Corporation, Massachusetts, USA; 17 Tokyo Foundation for Policy Research, Tokyo, Japan; 18 Department of Cardiovascular Medicine, Kyorin University Faculty of Medicine, Tokyo, Japan

**Keywords:** Palliative care, Cardiovascular diseases, Heart failure, Death, Surveys and questionnaires

## Abstract

**Background:**

Outcome measures during acute cardiovascular disease (CVD) phases, such as quality of death, have not been thoroughly evaluated. This is the first study that compared the family members’ perceptions of quality of death in deceased CVD patients and in deceased cancer patients using a bereaved family survey.

**Methods:**

Retrospectively sent questionnaire to consecutive family members of deceased patients with CVD from ten tertiary hospitals from October 2017 to August 2018. We used the short version of the Good Death Inventory (GDI) and assessed overall care satisfaction. Referencing the GDI, the quality of death was compared between CVD patients admitted to a non-palliative care unit (non-PCU) and cancer patients in palliative care units (PCU) and non-PCUs in the Japan Hospice and Palliative Care Evaluation Study (J-HOPE Study). Additionally, in the adjusted analysis, multivariable linear regression was performed for total GDI score adjusted by the patient and participant characteristics to estimate the difference between CVD and other patients.

**Results:**

Of the 243 bereaved family responses in agreement (response rate: 58.7%) for CVD patients, deceased patients comprised 133 (54.7%) men who were 80.2 ± 12.2 years old on admission. The GDI score among CVD patients (75.0 ± 15.7) was lower (worse) than that of cancer patients in the PCUs (80.2 ± 14.3), but higher than in non-PCUs (74.4 ± 15.2). After adjustment, the total GDI score for CVD patients was 7.10 points lower [95% CI: 5.22–8.97] than for cancer patients in PCUs and showed no significant differences compared with those in non-PCUs (estimates, 1.62; 95% CI [-0.46 to 5.22]).

**Conclusions:**

The quality of death perceived by bereaved family members among deceased acute CVD patients did not differ significantly from that of deceased cancer patients in general wards, however, was significantly lower than that of deceased cancer patients admitted in PCUs.

**Supplementary Information:**

The online version contains supplementary material available at 10.1186/s12904-024-01521-4.

## Introduction

Globally, an annual estimate of 20 million people requires palliative care [1]. Owing to current aging circumstances and advances in new invasive treatment modalities, palliative care for patients with non-cancer chronic diseases, especially cardiovascular disease (CVD), has received more attention [2]. Palliative care for CVD is complex because of the unpredictable disease trajectory, including acute cardiovascular events [3]. In contrast to cancer patients, the proportion of patients admitted emergently in acute care hospitals was higher in patients with CVD [4]. Although palliative care intervention for CVD in the chronic phase has been explored recently, palliative care for CVD in the acute phase has not been thoroughly evaluated [5–8].

Despite several debates about the outcome measures of palliative care, good death has long been considered one of the outcome measures of palliative care [9]. Because it is challenging to evaluate good death directly from patients, measures such as the bereaved family survey have been developed to evaluate the quality of death among patients [10, 11]. It is influenced by race/ethnicity and is usually adapted accordingly [12]. The Good Death Inventory (GDI) was developed and validated in Japan. It is characterized by including medical aspects (such as symptoms), the medical system, the place of treatment, and spiritual aspects, which was also validated in other Asian countries [13–16]. For cancer patients in general wards and palliative care units (PCU), nationwide surveys, such as the Japan Hospice and Palliative Care Evaluation Study (J-HOPE Study), have periodically been used to evaluate GDI.

We hypothesized that a bereaved family survey could capture the current status of palliative care in patients with acute CVD. In this study, we performed a bereaved family survey using a previously validated GDI for bereaved family members of acute CVD patients who had died in the hospital and compared the quality of death with bereaved family members of cancer patients to explore the target problems in clinical settings.

## Materials and methods

### Study designs, setting, and population

In this multicenter retrospective study, we enrolled deceased acute CVD patients from ten tertiary hospitals in Japan (S1 Table) admitted between January 2014 and December 2016 from Quality of PAlliative Care in the Heart Disease (Q-PACH) study, and deceased cancer patients registered in the J-HOPE3 study (S1 Figure). This work follows the guidelines of the STROBE initiative for cohort studies (S2 Table). Acute CVD patients were defined as those who were emergently hospitalized with a primary cardiac diagnosis of acute heart failure, acute coronary syndrome, acute limb ischemia, acute arrhythmia, venous thrombosis, or other acute cardiovascular conditions during the study period. The details of the primary cardiac diagnosis are listed in Table 1. Acute CVD patients were not admitted to palliative care units and hospices in Japan. Therefore, this study was limited to CVD patients admitted to university or tertiary referral hospitals, and the rest of study procedures were similar to the J-HOPE Study, except for patient selection [17, 18].

**Table 1:** Baseline demographics among each dataset

	CVD^1^		non-PCU^2^ Cancer	PCU Cancer
	*n* = 243		*n* = 682	*n* = 6397
***About patients***				
Patient age, y	80.2 ± 12.2		70.5 ± 11.5	73.9 ± 11.5
Patient gender, *n* (%)				
Male	133 (54.7)		412 (60.4)	3421 (53.5)
Primary cardiac diagnosis, n(%)		Primary site of cancer, n(%)		
Heart failure	146 (60.1)	Lung	135 (19.8)	1501 (23.5)
Acute coronary syndrome	26 (10.7)	Stomach	79 (11.6)	696 (10.9)
Out of hospital cardiac arrest	11 (4.5)	Colon	51 (7.5)	518 (8.1)
Acute/chronic limb ischemia	10 (4.1)	Rectum	32 (4.7)	276 (4.3)
Arrhythmia	8 (3.3)	Liver	29 (4.3)	284 (4.4)
Acute aortic disease	6 (2.5)	Gall bladder	36 (5.3)	294 (4.6)
Pulmonary embolism	2 (0.8)	Pancreas	64 (9.4)	628 (9.8)
Infectious disease including pneumonia	16 (6.6)	Esophagus	16 (2.3)	189 (3.0)
Others	18 (7.4)	Breast	54 (7.9)	300 (4.7)
-	-	Other	186 (27.3)	1711 (26.7)
***Participants (Bereaved family members)***				
Age, y	64.0 ± 11.8		61.5 ± 12	60.8 ± 12.1
Gender				
Male, n (%)	93 (38.3)		246 (36.1)	2217 (34.7)
*Missing*, n(%)	2 (0.8)		11 (1.6)	89 (1.4)
Relationship to decedent, n(%)				
Spouse	80 (32.9)		388 (56.9)	2664 (41.6)
Children	121 (49.8)		196 (28.7)	2507 (39.2)
Children-in-law	24 (9.9)		35 (5.1)	388 (6.1)
Parent	3 (1.2)		17 (2.5)	131 (2.0)
Sibling	6 (2.5)		31 (4.5)	431 (6.7)
Other	8 (3.3)		9 (1.3)	216 (3.4)

Total percentages for several categories do not reach 100% owing to missing values

1: CVD, cardiovascular disease

2: PCU, palliative care unit;

The J-HOPE study is a large-scale nationwide survey aimed at evaluating the quality of palliative care for cancer patients in Japan. It targets general hospitals, hospice and palliative care wards, clinics, and other healthcare facilities, using a bereaved family survey to comprehensively evaluate the quality of end-of-life care. The first J-HOPE study was conducted in 2007–2008, followed by the second J-HOPE2 study in 2010–2011, and the J-HOPE3 study in 2014. From the mentioned J-HOPE studies, the J-HOPE3 was utilized because the study date aligned with the commencement of this study. Of the 8097 effective replies of J-HOPE 3, we used data from 682 acute hospital cancer patients (non-PCU) and 6,397 PCU patients (excluding 1,018 home palliative care patients).

While this study involves merging distinct cohort data, it is important to note that this was possible due to the similar contextual backgrounds of the research participants, allowing for comparability based on temporal resemblance, geographical alignment, and uniformity of survey measurement items [19]. In this study, we conducted a bereaved family survey without considering the timing of death. Although recall bias is important, considering the ethical barriers to conducting bereaved family surveys in CVD patients from the perspective of the trade-off with sample size, we analyzed all applicable bereaved families. Regarding the sample size, it was difficult to make an a priori prediction because the design of a bereaved family survey targeting CVD patients is very challenging, and there were limited previous reports of GDI surveys targeting such a population. Considering that the overall response rate in the J-HOPE study was approximately 60%, we enrolled as many patients as possible to extract a sample size that could withstand a three-group comparison to clarify the differences in QOL between cancer patients and CVD patients.

### Criteria for selection of patients and bereaved family members

The inclusion and exclusion criteria for family members of cancer patients enrolled in J-HOPE have been described in detail in previous studies [20]. Details were collected for all CVD patients from ten tertiary hospitals admitted between January 2014 and December 2016 who met the following inclusion criteria: (a) patients who died at the hospital; (b) patients aged 20 years or older; and (c) bereaved family members aged 20 years or older. The exclusion criteria were as follows: (a) no identifiable address for the bereaved family members; (b) family members with severe psychological distress as determined by their primary physician; and (c) family members incapable of completing the self-reported questionnaire [17]. This study focused on GDI and overall care satisfaction to compare the quality of death between CVD and cancer. Family members who refused to participate were excluded after collecting the responses.

### Questionnaire sending protocol and consent obtaining

The set of paper copies of the questionnaires was sent to bereaved families from each participating institution, along with a letter explaining the survey, from July 2017 to August 2018. This wide range of sending schedules was due to differing timings of approval of each hospital’s institutional review board. Incentives to participate included a ballpoint pen in the envelope. The participants were asked to return the completed questionnaire to the secretariat office (St. Luke’s International Hospital) within two weeks. A month after the initial mail, non-responders were sent a reminder. In case of unwillingness to participate, they were asked to check a “no participation’’ box with the reason and return the incomplete questionnaire.

In this study, participant information was given and informed consent was asked to the families of CVD patients at the beginning of the questionnaire, and the patients’ families completing and submitting the questionnaire. As for the data from the 2014 J-HOPE3 study targeting cancer patients’ families, consent for data use had already been obtained from the patients’ families at the time of patient registration in the previous J-HOPE3 study, and new consent was obtained through an opt-out process [17, 18].

### Outcome measures and questionnaires

The primary outcomes were the total GDI score (continuous variable from 18 to 126), overall care satisfaction (satisfied or not), by bereaved family members, and the achievement (achieved or not) of the core ten attributes of the GDI by bereaved family members.

The questionnaires of this bereaved family survey is composed of the same content as previously used in the J-HOPE study, and common questionnaires, including the GDI [13, 17] and other popular validated questionnaires including, the Care Evaluation Scale (CES) [21, 22], Patient Health Questionnaire 9 (PHQ-9) [23], and the Brief Grief Questionnaire (BGQ) [24, 25], were collected as original forms in the survey. To maintain consistency with the questionnaire conditions in the previous J-HOPE3 study, we conducted a bereaved family survey consisting of the aforementioned questionnaires and utilized the “GDI” and “overall care satisfaction” from the survey. Regarding the questionnaires for GDI and overall care satisfaction, we used the exact same questionnaire content as in the previous J-HOPE3 study. The questionnaire comprised 15 pages (120–140 items in total) and took approximately 20–30 min to complete. Additionally, the participating institutions were requested to collect data on age, gender, and the chief cardiovascular diagnosis.

#### - good death inventory—short version

The GDI was originally developed for families of cancer patients and has demonstrated high reliability and validity. The total GDI score was calculated by summing the scores for all attributes. The total GDI score, which has been validated in previous reports to evaluate the degree of achievement of a good death from the perspective of bereaved family members in Japan, has been demonstrated to indicate the attainment of a good death when scores are high [26, 27]. Moreover, preliminary investigations of the research have also shown high reliability and validity when applied to families of non-cancer patients [13, 28]. We used the short version of GDI to measure whether patients experienced good death from the perspective of the bereaved family members. The original version of the GDI consists of ten core and eight optional domains and 54 attributes. The 10 core domains evaluate the attributes that Japanese people consistently rated as important, and the eight optional domains assess the attributes that are rated as important inconsistently and depend upon individual values [13, 17]. The short version comprised 18 representative items from each domain, and the validity and reliability of the scale were confirmed. The participants evaluated each attribute using a seven-point Likert-type scale (1 = absolutely disagree to 7 = absolutely agree).

#### - overall satisfaction

The question to the participants about their overall care satisfaction to examine concurrent validity was: “Overall, were you satisfied with the care in the hospital?” Using a six-point Likert scale, they answered from one (absolutely dissatisfied) to six (absolutely satisfied).

### Statistical methods

Continuous variables are presented as the mean ± standard deviation (SD), and categorical variables are presented as proportions (counts). Baseline covariate distributions were compared between the CVD, non-PCU cancer, and PCU cancer patients, using the Student’s t-test for continuous variables and one-way ANOVA for categorical variables.

For overall care satisfaction perceived by bereaved family members, ‘‘somewhat satisfied,’’ ‘‘satisfied,’’ and ‘‘absolutely satisfied’’ were collapsed into “satisfaction with overall care;” and for each component of GDI, ‘‘somewhat agree,’’ ‘‘agree,’’ and ‘‘absolutely agree’’ were collapsed into “achieved good death” to illustrate the distribution of scores and perform binary logistic regression later.

First, as an univariable model, GDI total scores and overall care satisfaction were compared among the bereaved families of CVD and cancer patients (in PCU and non-PCU), using a Kruskal-Wallis test to detect differences in median values across these three groups. Post-hoc pairwise comparisons were conducted using Dunn’s test with Bonferroni correction (p-value adjusted) to identify which groups differed significantly from each other.

Second, to estimate the difference of perceptions by bereaved families between CVD and cancer patients (in PCU and non-PCU), multivariable linear regression was performed for the total GDI score adjusted by covariates; the baseline patient characteristics (patient age, gender, disease duration, and physical status before admission) and baseline participant characteristics (participant age, gender, relationship to the patients, health status during the caregiving period, frequency of attending to the patient, and presence of other caregivers) [14].

Third, binary logistic regression models were performed for overall care satisfaction (“satisfied with overall care”) and for each component of GDI domains (“achieved good death” in core ten and optional eight attributes) perceived by bereaved family members to evaluate the impact of CVD on each GDI domains, adjusting the same covariates in the multivariable linear regression.

Furthermore, we performed sensitivity analyses by categorizing the patients into several cohorts to account for the unique trajectory of disease progression among CVD patients. In particular, since the responses to GDI, and overall care satisfaction may differ depending on the relationship between patients and the bereaved family, stratification by the target family was fundamentally important. Sensitivity analyses were conducted for patients who had received CVD/cancer treatment for over one year, patients with CVD limited to heart failure or cancer, patients with bereaved family members restricted to spouses or children, and an additional sensitive analysis focused on patients who had not received palliative care.

To confirm the robustness of the results, we performed propensity score matching using patient age and sex, and bereaved family member age and sex among the three groups: CVD, PCU cancer, and non-PCU cancer. Excluding two CVD patients with missing data on bereaved family members’ age, propensity scores were calculated and matching was performed for two comparisons: Bereaved family members of Cancer non-PCU vs. Cardiology, and Bereaved family members of Cancer PCU vs. Cardiology. Propensity scores were estimated using a non-parsimonious multivariable logistic regression model that included the variables Patients’ age, Patients’ sex, Families’ age, and Families’ sex. Matching was performed using the one-to-one matching protocol without replacement within a caliper width equal to 0.2 of the standard deviation of the logit of the propensity score. A total of 241 pairs of patients were successfully matched. Analyses were performed using R version 4.2.3 (R Foundation for Statistical Computing, Vienna, Austria), and a two-sided P-value < 0.05 threshold was used to determine statistical significance.

## Results

### Patients Flow of this study

A total of 907 patients with acute CVD died in the ten hospitals, of which 105 were excluded mainly due to being thought to be inappropriate by the chief attending physician (S2 Figure). Questionnaires were sent to 802 bereaved family members (88.4%), and out of them 112 (13.9%) were undeliverable due to incorrect addresses. Ultimately, questionnaires were sent to 690 bereaved family members (86%). Of the 690 questionnaires distributed, 405 were returned (58.7%) and 110 bereaved family members (27.1%) refused to participate (S1 Table). The reasons for non-participation were as follows: “It is hard to remember what happened when the patient died” [34 (30.9%)]; “I think the hospitalization period and the period of medical treatment at home are too short to be helpful” [25 (22.7%)]; “I do not have my thoughts in order, I do not want to be reminded of the incident” [21 (19.1%)]; and other (e.g. “Questionnaire is not appropriate;” [18 (16.4%)] (S3 Table).

### Baseline characteristics

Of the 295 valid responses from bereaved families of CVD patients, after excluding 52 questionnaires with missing GDI scores, 243 responses were analyzed. Table 1 compares the demographics of CVD patients and bereaved families with those of cancer patients. On admission, most CVD patients were male (54.7%) with an average age of 80.2 ± 12.2 years, significantly older than cancer patients. The most frequent primary diagnosis on admission was heart failure (146, 60.1%), followed by acute coronary syndrome (26, 10.7%). The participants were 64.0 ± 11.8 years old and mostly female (150; 61.7%). About half of them were children of deceased patients (49.8%) and one-third were spouses (32.9%), in contrast to cancer patients where the spouse was the chief participant.

Table 2 shows the questionnaire results regarding patient background by bereaved family members. Of the CVD patients, 60.9% were managed by a physician for more than three years and 16.9% for less than three months. Some were independent (34.2%) or required assistance (40.3%) before admission. Of the CVD patients, 13.6% received palliative care team approach (Involvement of a palliative care team in patient care); this was significantly lower than in cancer patients (53.4% of non-PCUs and 78.9% of PCUs). These features also contrast the acute CVD population to those in the cancer population.

**Table 2:** Background data of participants

	CVD^1^	non-PCU^2^ Cancer	PCU Cancer	*p*-value
	*n* = 243	*n* = 682	*n* = 6397	
***About patients***				
How long has the patient been seeing a doctor for CVD/cancer treatment?				
More than 3 years	148 (60.9)	186 (27.3)	1579 (24.7)	< 0.001
1–3 years	31 (12.8)	199 (29.2)	1992 (31.1)	
6 months to 1 year	13 (5.3)	107 (15.7)	1053 (16.5)	
3 to 6 months	7 (2.9)	77 (11.3)	855 (13.4)	
Less than 3 months	41 (16.9)	103 (15.1)	846 (13.2)	
How was the state of life just before admission to the PCU/hospital? Please select the closest one below.				
Life was independent	83 (34.2)	260 (38.1)	1392 (21.8)	< 0.001
Some assistance was required	98 (40.3)	277 (40.6)	2643 (41.3)	
Needed help in almost all cases	61 (25.1)	142 (20.8)	2330 (36.4)	
Did patient receive palliative care team approach?				
Yes	33 (13.6)	364 (53.4)	5045 (78.9)	< 0.001
No	158 (65.0)	195 (28.6)	860 (13.4)	
Unknown	52 (21.4)	123 (18.0)	492 (7.7)	
***About Responders (Bereaved family members)***				
Health status during caregiving period				0.001
Good	76 (31.3)	114 (16.7)	1366 (21.4)	
Moderate	114 (46.9)	375 (55.0)	3470 (54.2)	
Fair	41 (16.9)	145 (21.3)	1239 (19.4)	
Bad	11 (4.5)	41 (6.0)	273 (4.3)	
Frequency of attending patient (days/week)				< 0.001
Every day	140 (57.6)	474 (69.5)	4078 (63.7)	
4–6	35 (14.4)	96 (14.1)	986 (15.4)	
1–3	45 (18.5)	74 (10.9)	994 (15.5)	
None	22 (9.1)	32 (4.7)	288 (4.5)	
Presence of other caregivers				0.044
Present	156 (64.2)	494 (72.4)	4659 (72.8)	
Absent	85 (35.0)	181 (26.5)	1665 (26.0)	

Total percentages for several categories do not reach 100% owing to missing values

1: CVD, cardiovascular disease

2: PCU, palliative care unit;

### Comparisons of total GDI scores

**Fig. 1:**
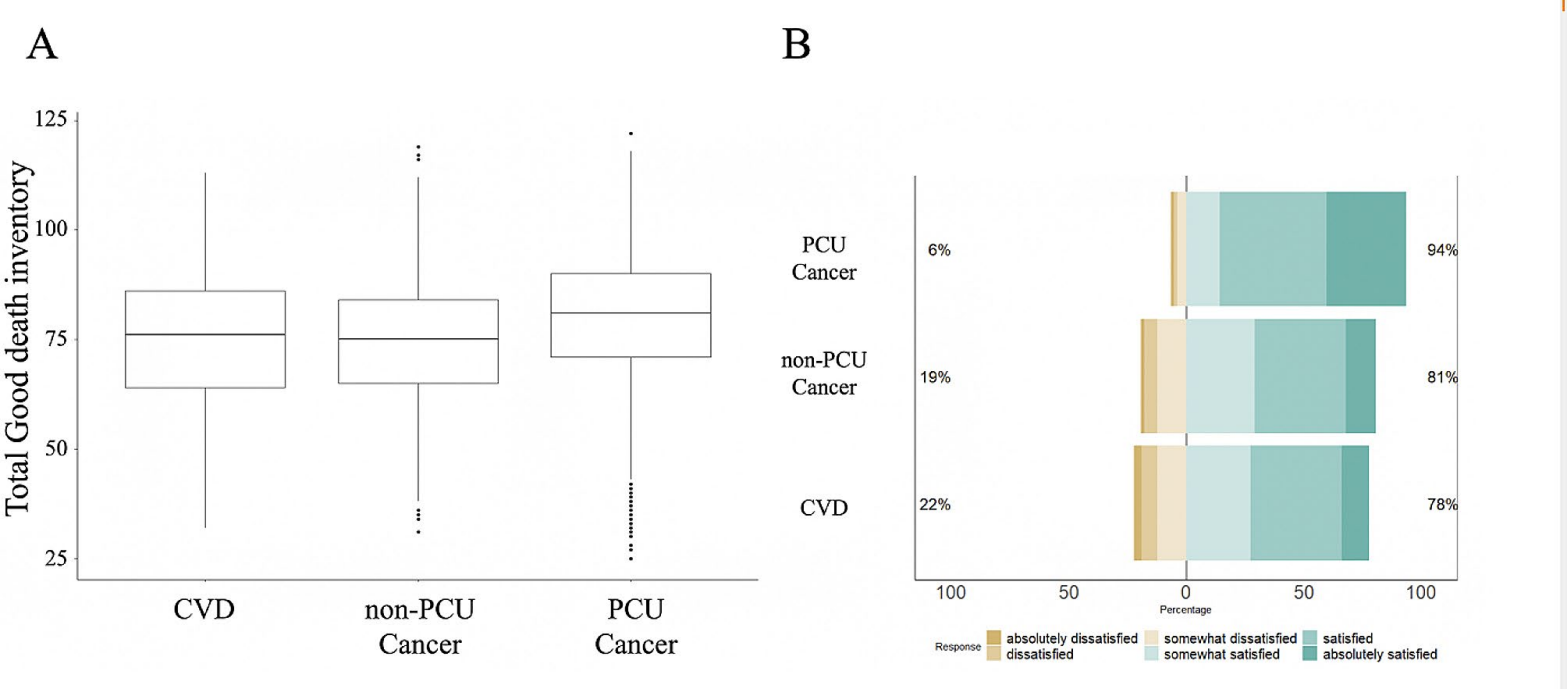
**A**. Box plot of the total good death inventory scores in each group. Caption: The upper whisker extended from the hinge to its largest value (no further than 1.5). × IQR, from the hinge. The lower whisker extends from the hinge to the smallest value at most 1.5. × IQR of the hinge. Data beyond the end of the whiskers are called “outlying” points and are plotted individually. **B**. Likert plot of the overall care evaluation scale for each population. Caption: The total sum of positive (“somewhat satisfied,” “satisfied,” “absolutely satisfied”) or negative (“absolutely dissatisfied,” “dissatisfied,” and “somewhat dissatisfied”) answering rate is shown on the left and right sides of the bar. IQR, Interquartile range; CVD, cardiovascular disease; PCU, palliative care unit

The total GDI score in bereaved family members of CVD patients (75.0 ± 15.7) was significantly lower than that in bereaved family members of PCU cancer patients (80.2 ± 14.3, *p* < .01) but similar to that in bereaved family members of non-PCU cancer patients (74.4 ± 15.2, *p* = .41; Fig. 1A). The overall care satisfaction scale in bereaved family members of CVD patients was 4.2 ± 1.2 (78% satisfied), which was also significantly lower than in bereaved family members of PCU cancer (5.0 ± 1.0, 94% satisfied, *p* < .01) and almost similar to bereaved family members of non-PCU cancer (4.4 ± 1.1, 81% satisfied, *p* = .76; Fig. 1B). After adjustment, Table 3 shows the GDI was 7.10 points higher [95% CI: 5.22–8.97] in bereaved family members of PCU cancer patients but showed no significant differences in bereaved family members of non-PCU cancer patients compared with bereaved family members of CVD patients (Estimates, 1.62; 95% CI [-0.46 to 5.22]). Table 3 also shows that after adjustment, the proportion of participants reporting satisfaction with the overall care they received was higher in both bereaved family members of PCU and non-PCU cancer patients than in bereaved family members of CVD patients (Adjusted odds ratio (aOR) 1.76 [95% CI, 1.18–2.61] and 5.63 [3.89–8.15], respectively).

**Table 3:** Multivariable analysis results

	non-PCU cancer (reference: CVD)			PCU^1^ cancer (reference: CVD^2^)		
	**Estimate**	**95%CI**	**p-value**	**Estimate**	**95%CI**	**p**
Total Good Death Inventory (score, continuous value)	1.62	[-0.46–5.22]	0.13	7.10	[5.22–8.97]	< 0.01
	**aOR** ^3^	**95%CI** ^4^	**p-value**	**aOR**	**95%CI**	**p**
Overall care satisfaction (satisfied, categorical value)	1.76	[1.18–2.61]	< 0.01	5.63	[3.89–8.15]	< 0.01
	non-PCU cancer (reference: CVD)			PCU cancer (reference: CVD)		
*Good Death Inventory*,* Core ten attributes(achieved*, categorical value*)*	**aOR**	**95%CI**	**p-value**	**aOR**	**95%CI**	**p**
Being free from physical distress	1.73	[1.26–2.37]	< 0.01	4.62	[3.46–6.17]	< 0.01
Being able to stay at one’s favorite place	1.22	[0.89–1.68]	0.22	2.13	[1.59–2.84]	< 0.01
Having some pleasure in daily life	1.21	[0.86–1.70]	0.28	2.03	[1.49–2.76]	< 0.01
Trusting physician	1.65	[1.17–2.34]	< 0.01	1.58	[1.16–2.14]	< 0.01
Not making trouble for others	0.68	[0.49–0.96]	0.03	0.82	[0.61–1.10]	0.18
Spending enough time with family	1.59	[1.16–2.19]	< 0.01	2.23	[1.67–2.97]	< 0.01
Being independent in daily activities	0.97	[0.68–1.40]	0.89	0.86	[0.62–1.20]	0.37
Living in calm circumstances	1.53	[1.11–2.11]	< 0.01	3.95	[2.95–5.30]	< 0.01
Being valued as a person	1.59	[1.04–2.43]	0.03	3.17	[2.16–4.68]	< 0.01
Feeling that life is complete	1.15	[0.82–1.59]	0.4	1.66	[1.23–2.23]	< 0.01
*Good Death Inventory*,* Option eight attributes (achieved)*						
Receiving sufficient treatment	1.25	[0.91–1.71]	0.17	1.55	[1.17–2.07]	< 0.01
Dying a natural death	1.83	[1.33–2.53]	< 0.01	3.62	[2.71–4.84]	< 0.01
Saying what wanted to tell loved ones	1.14	[0.83–1.56]	0.42	1.69	[1.28–2.25]	< 0.01
Knowing what to expect about future condition	1.52	[1.10–2.12]	0.01	1.92	[1.43–2.59]	< 0.01
Dying without awareness that one is dying	1.17	[0.80–1.71]	0.43	1.52	[1.08–2.13]	0.02
Not exposing physical and mental weakness to family	0.90	[0.62–1.30]	0.57	1.00	[0.72–1.38]	0.99
Feeling that life is worth living	1.18	[0.86–1.60]	0.31	1.15	[0.87–1.52]	0.33
Supported by religion	1.17	[0.79–1.75]	0.42	1.26	[0.88–1.80]	0.20

Each items are adjusted by covariates; the baseline patient characteristics (patient age, gender, disease duration, and physical status before admission) and baseline participant characteristics (participant age, gender, relationship to the patients, health status during the caregiving period, frequency of attending to the patient, and presence of other caregivers)

1: PCU, palliative care unit

2: CVD, cardiovascular disease

3: aOR, adjusted odds ratio

4: CI, confidence interval

### Comparison of achievement of each GDI component

**Fig. 2:**
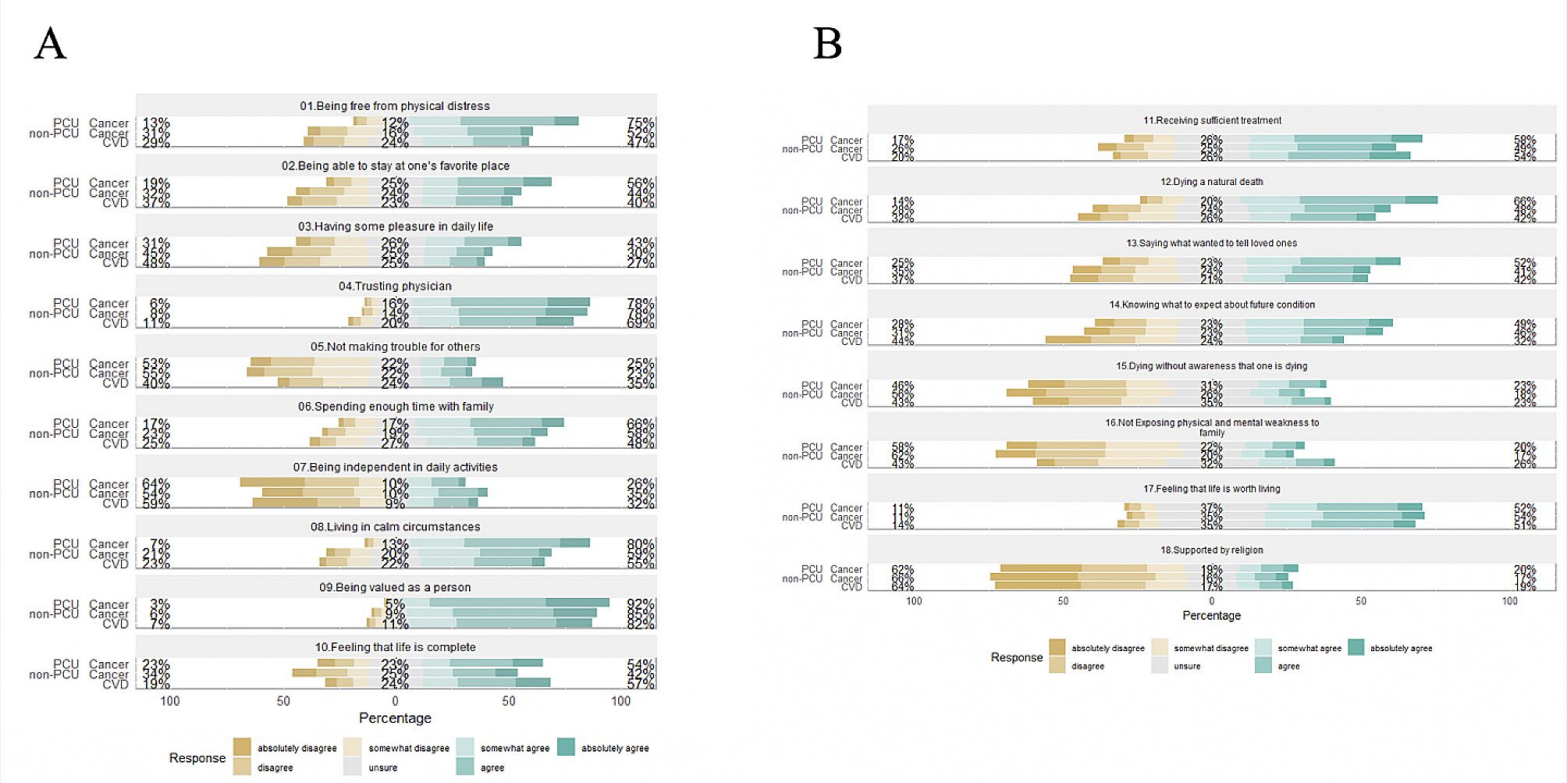
**A**. Likert plot of core ten attributes in the Good Death Inventory. **B**. Likert plot of option eight attributes in the Good Death Inventory. Caption: The total sum of positive (“somewhat agree,’’ ‘‘agree,’’ and ‘‘absolutely agree”) or negative (“somewhat disagree,’’ ‘‘disagree,’’ and ‘‘absolutely disagree) answering rate is shown in left and right of the bar. CVD, cardiovascular disease; PCU, palliative care unit

We also compared each GDI component (ten core attributes and eight option attributes) among these groups (Fig. 2A, B). “Achieved good death” for most items (both attributes sets) were higher (i.e., better quality of death) in bereaved family members of PCU cancer patients compared with bereaved family members of CVD patients, with some exceptions (“not making trouble for others” and “not exposing physical and mental weakness to family”). After adjustment, “being free from physical distress” (aOR: 4.62, [95% CI: 3.46–6.17]), “spending enough time with family” (aOR: 2.23, [95%CI: 1.67–2.97]), “living in calm circumstances” (aOR: 3.95, [95%CI: 2.95–5.30]), “being valued as a person” (aOR: 3.17, [95% CI: 2.16–4.68]), and “dying a natural death” (aOR: 3.62, [95% CI: 2.71–4.84]) were significantly better in PCU patients (Table 3).

After adjustment, the proportion of patients who experienced good death from the bereaved families’ perspective was significantly higher in bereaved family members of non-PCU cancer patients than bereaved family members of CVD patients in several components such as “being free from physical distress” (aOR: 1.73, [95% CI: 1.26–2.37]), “trusting physician” (aOR: 1.65, [95% CI: 1.17–2.34]), “spending enough time with family” (aOR: 1.59, [95% CI: 1.16–2.19]), “living in calm circumstances” (aOR: 1.53, [95% CI: 1.11–2.11]), “dying a natural death” (aOR: 1.83, [95% CI: 1.33–2.53]), and “knowing what to expect about future condition” (aOR: 1.52, [95% CI: 1.10–2.12]). However, the proportion of patients who achieved a good death based on “not making trouble for others” was significantly higher in the bereaved family members of CVD population than in the bereaved family members of PCU and non-PCU cancer populations.

### Sensitivity analysis and propensity score matching

Furthermore, we conducted sensitivity analyses across various subgroups (S4 Table), and we compared the tendencies of each component of the GDI across all 18 items and visualized the comparisons (S3 Figure). Sensitivity analyses of patients who had received CVD/cancer treatment for over one year, patients with CVD limited to heart failure and cancer, patients with bereaved family members limited to either spouses or children, and patients who had not received palliative care demonstrated that GDI scores were significantly higher among bereaved family members of PCU cancer patients compared to bereaved family members of CVD patients (Estimates: 5.61 points [95% CI: 3.39–7.83], 5.19 [95% CI: 2.85–7.53], 6.09 [95% CI: 2.87–9.31], 8.46 [95% CI: 5.75–11.2], 9.38 [95% CI: 6.78–12.0], respectively). Patient backgrounds after propensity score matching are shown in S5 Table. In this cohort as well, GDI scores were significantly higher in bereaved family members of PCU cancer patients compared to bereaved family members of CVD patients, demonstrating the robustness of the results (Estimates: 10.7 points [95% CI: 7.47–13.9]) (S6 Table). This trend is consistent in these sensitivity analyses, except for the sub-analysis of patients who did not receive palliative care team approach. Regarding overall care satisfaction, statistically significant lower levels were observed in all sensitivity analyses for bereaved family members of CVD patients compared to bereaved family members of PCU cancer patients.

## Discussion

To the best of our knowledge, this study is the first on the bereaved family members’ perspectives of quality of death in acute CVD patients compared with cancer patients in both PCU and non-PCU acute care hospitals, using the same bereaved family survey. The main findings were: (1) the GDI total score in bereaved family members of CVD patients was not different from that in the bereaved family members of non-PCU cancer population but significantly lower compared with the PCU cancer population; (2) the differences between GDI items and overall care satisfaction among the groups could indicate ways to improve CVD palliative care; environment and symptom control were essential, and “knowing future condition” in acute CVD is suboptimal from the bereaved families’ perspectives and is one of the clinically significant features to improve.

### GDI between acute CVD and cancer

Although previous studies have not directly compared the bereaved family members’ perspectives of good death between acute CVD and cancer patients, the impact of the place of death on a good death has been explored. Choi et al. [29] reported that quality of death and dying (QODD) scores were significantly lower in the intensive care unit than in the hospice. Several studies have also shown a lower quality of death in intensive care units and acute care settings than in hospices or palliative care facilities [30, 31]. In our study, the total GDI score by bereaved family members of CVD patients was significantly lower than that of bereaved family members of PCU cancer patients, but no significant differences were found between bereaved family members of cancer and bereaved family members of CVD in the non-PCU setting. The numerical differences in GDI scores have already been shown to correlate with satisfaction with end-of-life care, overall quality of death, and overall quality of life [15, 16]. GDI scores have demonstrated excellent structural validity and cross-cultural validity, and have been reported to provide the highest quality evidence supporting their use in assessing the quality of dying and death in Asian populations [32]. The result of this study could recognize the importance of the place of death, especially in palliative care units, as integral to the quality of death.

### Care satisfaction between acute CVD and cancer

Except for the validated quality of death measurements, such as QODD, subjective overall ratings have more frequently been adopted to evaluate palliative care. Ersek et al. [33] reported that hospice and palliative care patients showed higher family ratings of overall care quality than patients in acute or intensive care units. Our data showed significantly lower subjective overall care satisfaction in bereaved family members of CVD patients than in bereaved family members of cancer patients in PCUs. Additionally, it revealed that overall care satisfaction in bereaved family members of acute CVD patients was slightly but significantly lower than in bereaved family members of non-PCU cancer patients at acute hospitals after adjustment. Reports from the Swedish Registry of Palliative Care indicate that only 4.2% of heart failure patients received specialized palliative care, suggesting a particular inadequacy of palliative care in the last week of life [34]. It is reported that hospital care tends to focus more on treatment and life-saving measures, and there is an urgent need to improve the quality of life before death [34]. Considering our results, PCUs could offer better subjective care satisfaction, but further evaluation of subjective satisfaction for CVD and non-CVD patients is needed [35].

### GDI items and acute CVD

Several deficits in end-of-life care for the acute CVD population have been identified according to the analysis of each GDI component. First, differences in symptom management and environmental conditions during end-of-life care were apparent between CVD and cancer patients in PCUs. Considering “being free from physical distress,” 75%, 52%, and 47% of bereaved families of PCU cancer, non-PCU cancer, and CVD patients, respectively believed that the patient achieved a good death. As symptom management is an important quality indicator in acute CVD, education on symptom management is urgently needed [36]. The application of medical insurance was adapted for palliative care regarding heart failure in 2018 in Japan. The Japanese Heart Failure Society introduced a palliative care education program that included specific sessions for symptom management in 2019. These new implementations could improve the management of symptoms of　CVD in Japan.

Second, environmental management, another quality indicator, would also be essential to improve acute CVD care because of the lower achievement of a good death in “living in calm circumstances” and “dying a natural death.” The proportion of private rooms in PCUs is much higher than in general wards, which contributes to an improved environment and favorable circumstances [14]. These features could explain why our scores relating to environmental management in bereaved family members of PCU cancer patients were higher than those in bereaved family members of CVD patients.

### Predictability and acute CVD

The most challenging issue of palliative care for CVD is a different disease trajectory compared to cancer patients. A strength of this study is that it highlighted the challenges of introducing palliative care for CVD patients with unpredictable disease trajectories, and as a result, it became clear that the findings are relevant not only to acute CVD patients but also to a broader target population. Although several acute-phase prediction models for cardiovascular events have been developed, they cannot be applied well in clinical practice. The item score of “knowing what to expect about future condition” for bereaved family members of CVD patients was significantly lower than those for bereaved family members of cancer patients in PCU and non-PCU settings in this study. The gap between patient- and model-predicted life expectancy, especially among heart failure patients, has been described [37]. To improve the gap, advance care planning and communication between healthcare providers and patients have been focused on recently [38, 39]. A recent systematic review revealed that advance care planning increased documentation, such as advance directives, and reduced depression [8]. It could not increase prediction accuracy and reduce uncertainty about cardiovascular events but could offer better communication.

### A good death in CVD patients

Finally, only “not making trouble for others” in bereaved family members of CVD patients showed a significantly higher score than bereaved family members of cancer patients. Relieving burdens is one of the major domains for end-of-life quality [40]. The disease trajectory is different between cancer and CVD, especially regarding sudden death, which is more frequently observed with CVD and might impact the burden for others [41]. Although “not making trouble for others” is one of the most important good death components reflecting the Japanese cultural context, this result reminded us of whether we could use the same good death measures for both cancer and acute CVD settings in the modern era because good death could change over time [42, 43]. To clarify the definition of a “good death” for Japanese people, interviews with cancer patients and healthcare providers, as well as citizen surveys, have been conducted [42]. However, there has not been sufficient investigation in the field of cardiology. While healthcare providers have traditionally focused on physical symptoms, relationships with family and healthcare providers, as well as spiritual aspects, are being identified as important concepts [44]. The concept of a “good death” is multifaceted, addressing not only the physical and medical aspects of end-of-life care but also considering the emotional, social, and spiritual needs of the dying person and their family. It emphasizes the importance of human relationships, tranquility, respect, personal fulfillment, and the meaning of life, highlighting the importance of interventions by specialized palliative care teams [45]. As the demand for palliative care for CVD increases, the heart failure guidelines recommend Class I (Evidence and/or general agreement that a given procedure or treatment is useful and effective) for the introduction of palliative care for heart failure patients [46]. However, the introduction of palliative care for CVD is not simple due to its unpredictable course of exacerbations and remissions. In this study, it was found that palliative care for acute CVD has not been sufficiently considered compared to cancer patients, and its implementation rate in clinical settings is also not high. Given these challenges, there may be room to consider introducing palliative care for CVD not only in the chronic phase but also from the acute phase. Integrating the current results suggests that initiating advance care planning earlier for both CVD patients and cancer patients could potentially be an effective intervention for improving the quality of death for CVD patients. Similarly, in populations facing death, the quality of death in the PCU for cancer patients is significantly different, highlighting an issue that deserves attention. This suggests that we still need to work on intervening the quality of care. At the same time, we might need to rethink the good death according to the current clinical situation where we fronted many non-cancer palliative care, necessary patients.

### Strengths and limitations

The strengths of our study include the comparison of GDI perceived by bereaved family members between patients with acute CVD and cancer patients using the same bereaved family survey. Although our research sheds light on some critical points to improve CVD, this study has several limitations. Initially, it is important to note that the GDI score and Overall care satisfaction used in this study are merely proxy measures and do not necessarily accurately reflect the quality of death of patients. Since the weighting of each item largely depends on individual values, caution should be exercised in uniform interpretation. However, considering the difficulty of evaluating the quality of death using objective measures, the GDI has undergone sufficient validation as a scale for evaluating death from the perspective of bereaved families in previous studies, making it possible to obtain a certain level of reliability. Second, we have only a small number of survey results compared with previous data [47]. Third, the duration of this study was longer than that of the J-HOPE 3 study. The difficulty of the questionnaire for bereaved families of acute CVD patients prevented the institutional review board from readily accepting this survey, thus resulting in a delay of approximately a year. The varying timing of when the questionnaires were sent to each bereaved family member is an important limitation, as it may have introduced recall bias. Fourth, the primary attending physician associated with this study denied sending questionnaires to 11.6% of patients (S2 Figure), which might have resulted in selection bias to improve the total score in acute CVD patients. A low response rate increases the risk of non-response bias, where the characteristics and experiences of those who respond to the survey may differ significantly from those who do not respond. For example, those who had more positive experiences may be more likely to respond, leading to an overestimation of the quality of care. Further, the small sample size due to the low response rate could have caused the apparent lack of differences between CVD and non-PCU cancer patients. Further, the quality of death was higher in PCU cancer patients, but the difference between non-PCU cancer and CVD patients should be evaluated using other datasets. Although our data highlighted some essential differences between them, further investigation in other datasets is needed. Fifth, the selection of patient cohorts presents an essential selection bias, making it challenging to compare the quality of palliative care due to the unpredictable trajectories CVD patients follow. In cases of acute CVD, acceptance of mortality is more challenging, and it is less straightforward to determine a sufficient time frame for families to become aware of impending death compared to cancer. The unique nature of the trajectory of acute CVD underscores the importance of identifying areas for improvement in the quality of this end-of-life experience. In this study, by conducting sensitivity analyses not only based on the type of disease but also the duration of treatment, we successfully compared patients who have undergone CVD or cancer treatment for over a year, further justifying the design of this study. Sixth, this study evaluates the quality of death as perceived by the family members, not the patients themselves. Therefore, the relationship between the bereaved family and the patient is crucial. To address this limitation to the fullest extent possible, we performed stratification based on the relationship and confirmed the robustness of the results, which were consistent across each subgroup. However, data on the objective distance from the patient, such as cohabitation status, were not available. While it is naturally expected that patients would be able to accurately assess the quality of their own death, due to the challenges in measurement, research related to end-of-life care, including the QODD scale evaluated abroad, primarily revolves around family care, and reports concerning patients’ experiences of a death are scarce. Given the difficulty in assessing the quality of death directly from patients themselves, further research into appropriate indicators as alternative variables is anticipated. Seventh, in the methodology of this study, although cancer patients (PCU and non-PCU) are set as a control group for acute CVD, strictly speaking, a comparison with acute cancer patients would be preferable. In the current cohort, data on acute hospitalizations for cancer due to acute complications were not available, making the above comparison difficult in this study. However, due to the unique trajectory of CVD leading to death, unlike cancer, most deaths occur during an acute course. In evaluating the quality of death, it is important to first confirm the differences in comparison with the overall cohort of cancer patients as a first step. As a next research possibility, it is necessary to suggest conducting a comparison with acute cancer patients to further clarify the quality of death in acute CVD. Lastly, it is important to acknowledge that the questionnaires from family members in this study were directly obtained from them and might not entirely reflect the patients’ actual experiences. For example, it is unclear whether patients with heart failure whose families reported them to their cardiologist as having been there for more than three years were truly continuing to see their cardiologist outpatient only for heart failure or not. However, by conducting surveys with the closest relatives, this study utilized data that is considered the most reliable alternative when compared to patient self-reported data, enhancing the credibility of the obtained patient information.

## Conclusions

This is the first study on the quality of death in patients with acute CVD compared with cancer patients using the bereaved family survey, including GDI and overall satisfaction. Notably, there were no significant differences in GDI scores between non-PCU cancer and CVD groups. However, the GDI score in PCU cancer patients was significantly higher than that in CVD patients. The results also revealed the need to improve palliative care for acute CVD patients. Cardiologists should consider several components to achieve better death, such as symptom management, environmental control such as palliative care units, and effective communications considering uncertainty. We should continue to discuss the appropriate manner of palliative care, especially for acute CVD.

### Electronic supplementary material

Below is the link to the electronic supplementary material.

Supplementary Material 1

Supplementary Material 2

Supplementary Material 3

Supplementary Material 4

Supplementary Material 5

Supplementary Material 6

Supplementary Material 7

Supplementary Material 8

Supplementary Material 9

## Data Availability

The datasets used and/or analyzed during the current study available from the corresponding author on reasonable request. Additional information regarding the findings presented can be requested from the corresponding author.
