# Supplementary material for: Bereaved family members’ perspectives on quality of death in deceased acute cardiovascular disease patients compared with cancer patients – a comparison of the J-HOPE3 study and the quality of palliative care in heart disease (Q-PACH) study
Source: BMC Palliat Care. 2024 Jul 26;23:188. doi: 10.1186/s12904-024-01521-4 (PMC11282702; doi:10.1186/s12904-024-01521-4)
Supplement: Supplementary file 1 — Supplementary Material 1 [file 12904_2024_1521_MOESM1_ESM.docx]

S1 Table: All acute cardiovascular patients from ten tertiary hospitals

|  | 1st  sending | 2nd reminder | Total patients died during the study period | Questionnaire  sent | Successfully sent | Questionnaire returned |
| --- | --- | --- | --- | --- | --- | --- |
| St. Luke’s International Hospital | 2017/7/12 | 2017/9/2 | 83 | 83 | 66 | 43 |
| Keio University Hospital | 2018/1/31 | 2018/3/8 | 76 | 70 | 57 | 36 |
| Tokyo Medical University Ibaraki Medical Center | 2017/11/22 | 2018/1/31 | 92 | 92 | 90 | 58 |
| Mito Saiseikai General Hospital | 2017/12/20 | 2018/2/7 | 160 | 143 | 129 | 81 |
| Tokyo Saiseikai Central Hospital | 2018/3/2 | 2018/5/7 | 60 | 26 | 24 | 7 |
| Kobe City Medical Center General Hospital | 2018/1/17 | 2018/3/5 | 132 | 132 | 111 | 63 |
| Kurume University Hospital | 2018/5/14 | 2018/6/25 | 65 | 65 | 53 | 34 |
| Tohoku University Hospital | 2018/5/2 | 2018/6/28 | 48 | 48 | 39 | 23 |
| Tokai University Hospital | 2018/6/12 | 2018/8/2 | 137 | 104 | 91 | 45 |
| Kyoto University Hospital | 2018/8/17 | 2018/9/19 | 54 | 39 | 30 | 15 |
