# Supplementary material for: Bereaved family members’ perspectives on quality of death in deceased acute cardiovascular disease patients compared with cancer patients – a comparison of the J-HOPE3 study and the quality of palliative care in heart disease (Q-PACH) study
Source: BMC Palliat Care. 2024 Jul 26;23:188. doi: 10.1186/s12904-024-01521-4 (PMC11282702; doi:10.1186/s12904-024-01521-4)
Supplement: Supplementary file 3 — Supplementary Material 3 [file 12904_2024_1521_MOESM3_ESM.docx]

S3 Table: The reasons for refusing participation

|  | n=110 |
| --- | --- |
| It is hard to remember what happened when the patient died | 34(30.9) |
| I think the hospitalization period and medical treatment period at home are too short to be helpful | 25(22.7) |
| I do not have my thoughts in order, I do not want to be reminded of the incident | 21(19.1) |
| I am not sure what the patient was like at the time | 15(13.6) |
| I am not good at answering questionnaires | 11(10) |
| I think that answering this questionnaire will not help me in the future | 8(7.3) |
| Dissatisfied with the hospital where the patient died or the home care received | 7(6.4) |
| I cannot answer because of poor eyesight, hand condition, physical condition, etc. | 7(6.4) |
| I do not have time to answer | 2(1.8) |
| I think that the facility will know what kind of answers the individual has made | 0(0) |
| Other (e.g., Question is not appropriate) | 18(16.4) |
