# Supplementary material for: Bereaved family members’ perspectives on quality of death in deceased acute cardiovascular disease patients compared with cancer patients – a comparison of the J-HOPE3 study and the quality of palliative care in heart disease (Q-PACH) study
Source: BMC Palliat Care. 2024 Jul 26;23:188. doi: 10.1186/s12904-024-01521-4 (PMC11282702; doi:10.1186/s12904-024-01521-4)
Supplement: Supplementary file 4 — Supplementary Material 4 [file 12904_2024_1521_MOESM4_ESM.docx]

**S4 Table.** The result of sensitivity analysis regarding the multivariable analysis

| ***Multivariable analysis comparing among patients who have seeing a doctor for CVD/cancer treatment more than 1 year*** | | | | | | | | | | | | | | | | | | | | |
| --- | --- | --- | --- | --- | --- | --- | --- | --- | --- | --- | --- | --- | --- | --- | --- | --- | --- | --- | --- | --- |
|  | CVD^1^ | | | | | | non-PCU^2^  Cancer | | | | | | | | | PCU  Cancer | | | | |
| Number of the patients, No (%) | 179 (73.7) | | | | | | 386 (56.6) | | | | | | | | | 3571 (55.8) | | | | |
|  | non-PCU cancer (reference: CVD) | | | | | | | | | | | PCU cancer (reference: CVD) | | | | | | | | |
|  | **Estimate** | | **95%CI** | | | | | **p-value** | | | | **Estimate** | | | | | **95%CI** | | | **p** |
| Total Good Death Inventory (score) | 0.73 | | [-1.80–3.27] | | | | | 0.57 | | | | 5.61 | | | | | [3.39–7.83] | | | <.01 |
|  | **OR**^3^ | | **95%CI**^4^ | | | | | **p-value** | | | | **OR** | | | | | **95%CI** | | | **p** |
| Overall care satisfaction (satisfied) | 1.80 | | [1.10–2.95] | | | | | 0.02 | | | | 5.52 | | | | | [3.52–8.66] | | | <.01 |
| ***Multivariable analysis comparing among heart failure patients and non-PCU/PCU cancer patients*** | | | | | | | | | | | | | | | | | | | | |
|  | CVD (HF^5^) | | | | | | non-PCU  Cancer | | | | | | | PCU  Cancer | | | | | | |
| Number of the patients, No (%) | 146 (60.1) | | | | | | 682 (100) | | | | | | | 6397 (100) | | | | | | |
|  | non-PCU cancer (reference: CVD) | | | | | | | | | | PCU cancer (reference: CVD) | | | | | | | | | |
|  | **Estimate** | | | **95%CI** | | | | **p-value** | | | **Estimate** | | | | **95%CI** | | | | **p** | |
| Total Good Death Inventory (score) | -0.31 | | | [-2.83–2.21] | | | | 0.81 | | | 5.19 | | | | [2.85–7.53] | | | | <.01 | |
|  | **OR**^3^ | | | **95%CI**^4^ | | | | **p-value** | | | **OR** | | | | **95%CI** | | | | **p** | |
| Overall care satisfaction (satisfied) | 1.55 | | | [0.96–2.51] | | | | 0.07 | | | 5.03 | | | | [1.07–2.29] | | | | 0.02 | |
| ***Multivariable analysis comparing among patients whose participants (Bereaved family members) were spouse*** | | | | | | | | | | | | | | | | | | | | |
|  | | CVD | | | | | non-PCU  Cancer | | | | | | | | PCU  Cancer | | | | | |
| Number of the patients, No (%) | | 80 (32.9) | | | | | 388 (56.9) | | | | | | | | 2664 (41.6) | | | | | |
|  | | non-PCU cancer (reference: CVD) | | | | | | | | | PCU cancer (reference: CVD) | | | | | | | | | |
|  | | **Estimate** | | | **95%CI** | | | | **p-value** | | **Estimate** | | | | | **95%CI** | | | | **p** |
| Total Good Death Inventory (score) | | 2.21 | | | [-1.20–5.62] | | | | 0.20 | | 6.09 | | | | | [2.87–9.31] | | | | <.01 |
|  | | **OR**^3^ | | | **95%CI**^4^ | | | | **p-value** | | **OR** | | | | | **95%CI** | | | | **p** |
| Overall care satisfaction (satisfied) | | 2.77 | | | [1.51–5.08] | | | | <.01 | | 6.55 | | | | | [3.67–11.7] | | | | <.01 |
| ***Multivariable analysis comparing among patients whose participants (Bereaved family members) were children*** | | | | | | | | | | | | | | | | | | | | |
|  | CVD | | | | | non-PCU  Cancer | | | | | | | | | PCU  Cancer | | | | | |
| Number of the patients, No (%) | 121 (49.8) | | | | | 196 (28.7) | | | | | | | | | 2507 (39.2) | | | | | |
|  | non-PCU cancer (reference: CVD) | | | | | | | | | | PCU cancer (reference: CVD) | | | | | | | | | |
|  | **Estimate** | | | **95%CI** | | | | **p-value** | | | **Estimate** | | | | | **95%CI** | | | | **p** |
| Total Good Death Inventory (score) | 0.97 | | | [-2.27–4.22] | | | | 0.56 | | | 8.46 | | | | | [5.75–11.2] | | | | <.01 |
|  | **OR**^3^ | | | **95%CI**^4^ | | | | **p-value** | | | **OR** | | | | | **95%CI** | | | | **p** |
| Overall care satisfaction (satisfied) | 0.91 | | | [0.49–1.69] | | | | 0.76 | | | 4.68 | | | | | 2.64–8.30] | | | | <.01 |
| ***Multivariable analysis comparing among patients who had not received palliative care team approach*** | | | | | | | | | | | | | | | | | | | | |
|  | CVD | | | | | | non-PCU  Cancer | | | | | | PCU  Cancer | | | | | | | |
| Number of the patients, No (%) | 158 (65.0) | | | | | | 195 (28.6) | | | | | | 860 (13.4) | | | | | | | |
|  | non-PCU cancer (reference: CVD) | | | | | | | | | PCU cancer (reference: CVD) | | | | | | | | | | |
|  | **Estimate** | | | **95%CI** | | | | **p-value** | | **Estimate** | | | | **95%CI** | | | | **p** | | |
| Total Good Death Inventory (score) | 3.30 | | | [0.14–6.46] | | | | 0.04 | | 9.38 | | | | [6.78–12.0] | | | | <.01 | | |
|  | **OR**^3^ | | | **95%CI**^4^ | | | | **p-value** | | **OR** | | | | **95%CI** | | | | **p** | | |
| Overall care satisfaction (satisfied) | 2.24 | | | [1.23–4.06] | | | | <.01 | | 10.55 | | | | [6.00–18.6] | | | | <.01 | | |

1: CVD, cardiovascular disease

2: PCU, palliative care unit

3: OR, odds ratio

4: CI, confidence interval

5: HF, heart failure
