# Supplementary material for: Bereaved family members’ perspectives on quality of death in deceased acute cardiovascular disease patients compared with cancer patients – a comparison of the J-HOPE3 study and the quality of palliative care in heart disease (Q-PACH) study
Source: BMC Palliat Care. 2024 Jul 26;23:188. doi: 10.1186/s12904-024-01521-4 (PMC11282702; doi:10.1186/s12904-024-01521-4)
Supplement: Supplementary file 5 — Supplementary Material 5 [file 12904_2024_1521_MOESM5_ESM.docx]

**S5 table.** Baseline demographics and outcomes among each dataset, after propensity score matching with age and sex of patients and participants.

|  | CVD^1^ | non-PCU^2^  Cancer | PCU  Cancer | p-value |
| --- | --- | --- | --- | --- |
|  | n=241 | n=241 | n=241 |  |
| ***About patients*** | | | |  |
| Patient age, y | 80.2 ± 12.3 | 80.5 ± 11.81 | 78.6 ± 10.4 | 0.165 |
| Patient gender, n (%) |  |  |  |  |
| Male | 131 (54.4) | 134 (55.6) | 139 (57.7) | 0.76 |
| ***Participants (Bereaved family members)*** | | | |  |
| Age, y | 64.0 ± 11.8 | 62.9 ± 12.5 | 63.3 ± 12.4 | 0.64 |
| Gender |  |  |  |  |
| Male, n (%) | 93 (38.6) | 93 (38.6) | 89 (36.9) | 0.71 |
| Relationship to decedent, n(%) |  |  |  | 0.016 |
| Spouse | 80 (33.2) | 73 (30.3) | 103 (42.7) |  |
| Children | 120 (49.8) | 130 (53.9) | 103 (42.7) |  |
| Children-in-law | 24 (10.0) | 10 (4.1) | 22 (9.1) |  |
| Parent | 3 (1.2) | 5 (2.1) | 4 (1.7) |  |
| Sibling | 6 (2.5) | 10 (4.1) | 3 (1.2) |  |
| Other | 8 (3.3) | 10 (4.1) | 4 (1.7) |  |
| ***About patients*** | | | | |
| How long has the patient been seeing a doctor for CVD/cancer treatment? | | | | |
| More than 3 years | 146 (60.6) | 44 (18.3) | 62 (25.7) | <0.001 |
| 1–3 years | 31 (12.9) | 71 (29.5) | 68 (28.2) |  |
| 6 months to 1 year | 13 (5.4) | 46 (19.1) | 43 (17.8) |  |
| 3 to 6 months | 7 (2.9) | 26 (10.8) | 27 (11.2) |  |
| Less than 3 months | 41 (17.0) | 52 (21.6) | 38 (15.8) |  |
| How was the state of life just before admission to the PCU/hospital? Please select the closest one below. | | | | |
| Life was independent | 81 (33.6) | 59 (24.5) | 80 (33.2) | 0.013 |
| Some assistance was required | 98 (40.7) | 88 (36.5) | 101 (41.9) |  |
| Needed help in almost all cases | 61 (25.3) | 93 (38.6) | 60 (24.9) |  |
| Did patient receive palliative care team approach? | | | | |
| Yes | 33 (13.7) | 172 (71.4) | 118 (49.0) | <0.001 |
| No | 156 (64.7) | 47 (19.5) | 90 (37.3) |  |
| Unknown | 52 (21.6) | 22 (9.1) | 33 (13.7) |  |
| ***About Responders (Bereaved family members)*** | | | | |
| Health status during caregiving period |  |  |  | 0.060 |
| Good | 75 (31.1) | 58 (24.1) | 45 (18.7) |  |
| Moderate | 114 (47.3) | 132 (54.8) | 141 (58.5) |  |
| Fair | 41 (17.0) | 42 (17.4) | 48 (19.9) |  |
| Bad | 11 (4.6) | 9 (3.7) | 7 (2.9) |  |
| Frequency of attending patient (days/week) |  |  |  | 0.024 |
| Every day | 140 (58.1) | 152 (63.1) | 167 (69.3) |  |
| 4–6 | 35 (14.5) | 31 (12.9) | 38 (15.8) |  |
| 1–3 | 45 (18.7) | 47 (19.5) | 25 (10.4) |  |
| None | 21 (8.7) | 10 (4.1) | 10 (4.1) |  |
| Presence of other caregivers |  |  |  | 0.003 |
| Present | 155 (64.3) | 191 (79.3) | 183 (75.9) |  |
| Absent | 85 (35.3) | 49 (20.3) | 58 (24.1) |  |
