# Supplementary figures and images for: Bereaved family members’ perspectives on quality of death in deceased acute cardiovascular disease patients compared with cancer patients – a comparison of the J-HOPE3 study and the quality of palliative care in heart disease (Q-PACH) study
Source: BMC Palliat Care. 2024 Jul 26;23:188. doi: 10.1186/s12904-024-01521-4 (PMC11282702; doi:10.1186/s12904-024-01521-4)

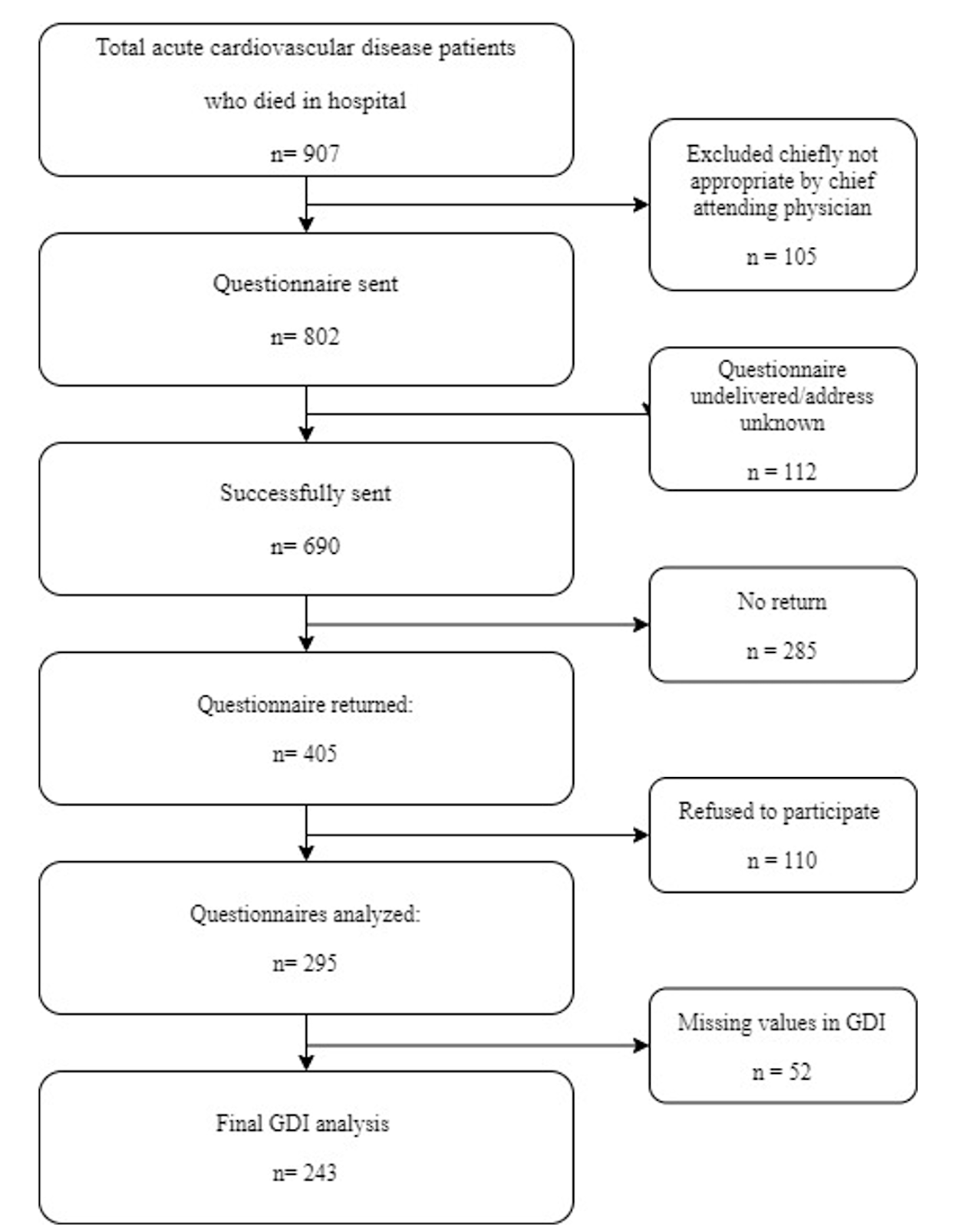

Supplement: Supplementary file 6 — Supplementary Material 6 [file 12904_2024_1521_MOESM6_ESM.png]

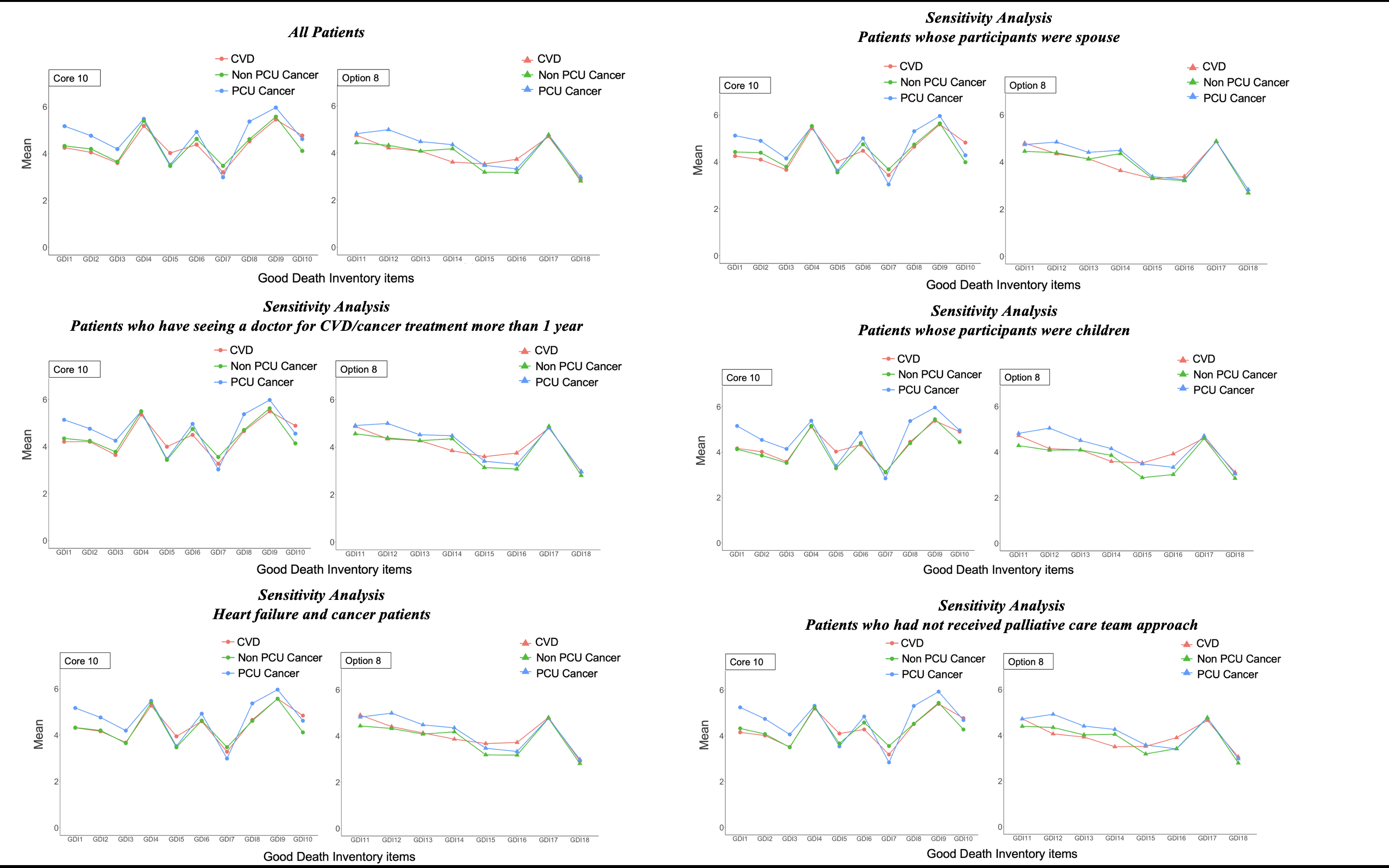

Supplement: Supplementary file 7 — Supplementary Material 7 [file 12904_2024_1521_MOESM7_ESM.png]

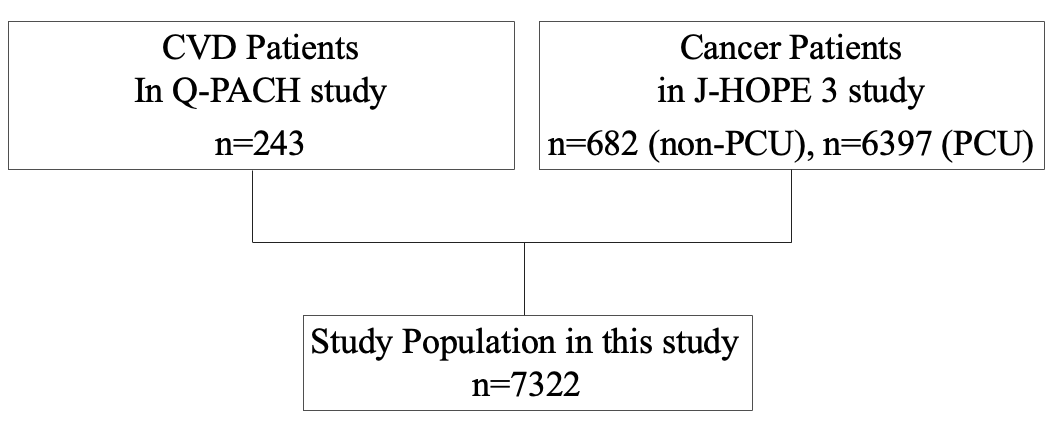

Supplement: Supplementary file 9 — Supplementary Material 9 [file 12904_2024_1521_MOESM9_ESM.png]
