# Supplementary material for: Bereaved family members’ perspectives on quality of death in deceased acute cardiovascular disease patients compared with cancer patients – a comparison of the J-HOPE3 study and the quality of palliative care in heart disease (Q-PACH) study
Source: BMC Palliat Care. 2024 Jul 26;23:188. doi: 10.1186/s12904-024-01521-4 (PMC11282702; doi:10.1186/s12904-024-01521-4)
Supplement: Supplementary file 8 — Supplementary Material 8 [file 12904_2024_1521_MOESM8_ESM.docx]

**S6 table.** The result of the multivariable analysis after propensity score matching

|  | non-PCU cancer (reference: CVD) | | | PCU cancer (reference: CVD) | | |
| --- | --- | --- | --- | --- | --- | --- |
|  | **Estimate** | **95%CI** | **p-value** | **Estimate** | **95%CI** | **p** |
| Total Good Death Inventory (score) | 3.28 | [0.31–6.25] | 0.03 | 10.7 | [7.47–13.9] | <.01 |
|  | **OR**^3^ | **95%CI**^4^ | **p-value** | **OR** | **95%CI** | **p** |
| Overall care satisfaction (satisfied) | 1.55 | [0.89–2.68] | 0.12 | 8.96 | [3.76–21.3] | <.01 |
